# Pediatric Osteochondrolipoma of the First Metacarpal: A Case Report and Literature Review

**DOI:** 10.1155/cro/9611206

**Published:** 2026-11-04

**Authors:** J. Graham Andre, Courtney Schneidau, Joseph Chang, Christopher Stutz

**Affiliations:** ^1^ Scottish Rite for Children Dallas Texas USA

## Abstract

**Introduction:**

Osteochondrolipoma (OCL) is a rare benign soft tissue neoplasm composed of mature adipose, cartilaginous, and osseous tissue. Reports in the pediatric population are exceedingly uncommon, and the lesion is seldom included in the differential diagnosis of a hand mass in a child. We report an OCL arising adjacent to the first metacarpal in a 9‐year‐old male.

**Case Presentation:**

A 9‐year‐old male presented with a firm, nonmobile, nontender mass overlying the dorsoradial aspect of the left first metacarpal. Radiographic evaluation demonstrated an ovoid soft tissue density with irregular central calcification. MRI demonstrated a well‐circumscribed mass with a thin hypointense capsule abutting the dorsal cortex of the first metacarpal, containing scattered foci of T1 hyperintense macroscopic fat. The mass was juxtacortical at the time of excision, in contact with the dorsal periosteum but without continuity with the underlying cortex. Histologic evaluation demonstrated mature adipose, cartilaginous, and osseous tissue, establishing the diagnosis of OCL.

**Conclusion:**

Although rare, OCL should be included in the differential diagnosis for a slow‐growing, painless soft tissue mass in pediatric patients of all age groups. In this case, complete marginal excision resulted in an uncomplicated recovery with no evidence of recurrence at 10 weeks postoperatively; however, conclusions regarding long‐term outcomes cannot be drawn from a single case with limited follow‐up.

## 1. Introduction

Lipoma is one of the most common benign neoplasms of soft tissue, occurring throughout the body with peak incidence in the fifth through seventh decades of life. Osteochondrolipoma (OCL) is a rare variant composed of adipocyte, chondrocyte, and osteocyte differentiation. This tumor occurs predominantly in adult populations, with a reported mean patient age in the sixth decade, and has been described in the maxillofacial region, the trunk, and the extremities [1, 2]. Reports in children are exceedingly rare, and OCL is seldom considered in the differential diagnosis of a hand mass in a skeletally immature patient. We present a case of OCL of the thumb metacarpal in a 9‐year‐old male and review the relevant literature.

## 2. Statement of Informed Consent

The patient and family were informed and agreed that data concerning this case would be submitted for publication.

## 3. Case Presentation

A 9‐year‐old right‐hand‐dominant male presented to our clinic for evaluation of a mass overlying the left thumb. The exact duration was unknown; however, the patient reported it had been present for over a year. It was first brought to the attention of medical practitioners after the patient fell on the playground and jammed his thumb. Physical examination of the left hand and wrist revealed a firm, nonmobile, nontender mass at the dorsal radial border of the thenar eminence (Figure 1). Thumb extension at the carpometacarpal joint was limited relative to the contralateral side; this represented a limitation of motion rather than a fixed deformity. There was no instability of the metacarpophalangeal joint to radial or ulnar stress, and the mass did not transilluminate.

**Figure 1:**
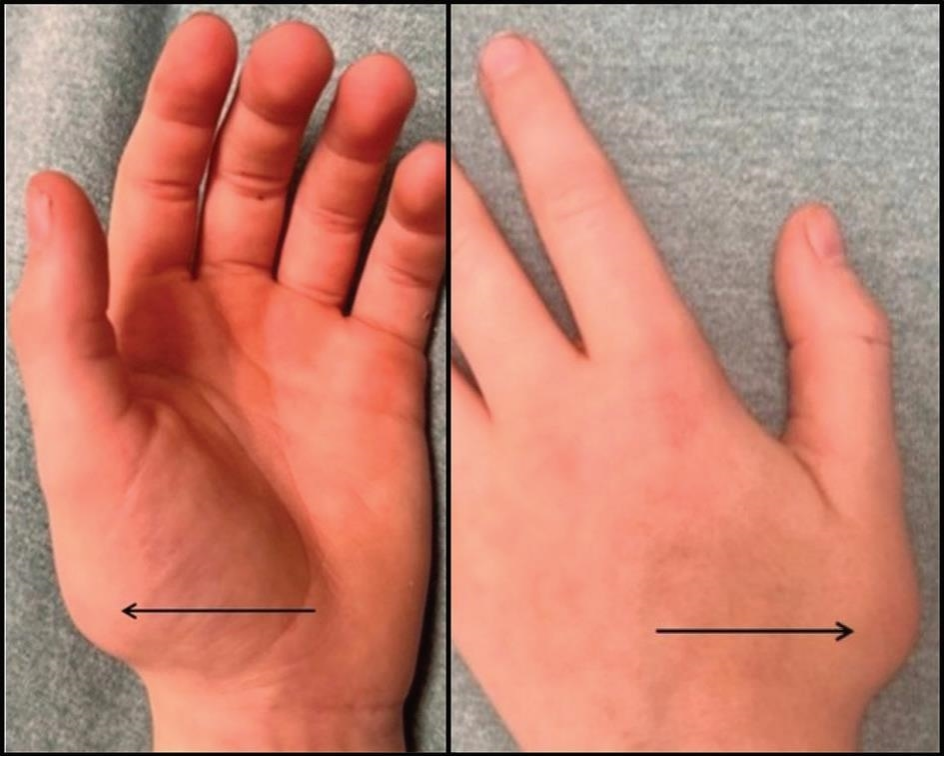
Clinical photographs of the left hand demonstrating a mass over the dorsoradial aspect of the thumb metacarpal (arrow).

Radiographic evaluation demonstrated an ovoid soft tissue density with irregular central calcification at the dorsal radial aspect of the proximal first metacarpal (Figure 2). MRI revealed an ovoid, well‐circumscribed mass with a thin hypointense capsule abutting the dorsal margin of the proximal first metacarpal, deep to the extensor pollicis longus tendon. Mild concave remodeling of the dorsal cortical margin of the first metacarpal was noted (Figure 3A), a finding consistent with a slow‐growing, long‐standing lesion. The mass demonstrated heterogeneous signal on fluid‐sensitive short tau inversion recovery (STIR) sequences. On coronal gradient‐echo (GRE) imaging, lobular central hypointensity corresponded to the calcification identified on plain radiography (Figure 3B). Scattered foci of T1 hyperintense macroscopic fat were present within the mass (Figure 3C), indicating a lipomatous component. There was no evidence of local invasion or reactive marrow edema in the adjacent bone. Because the preoperative assessment was based on imaging alone, the differential diagnosis remained broad and included tenosynovial giant cell tumor, fibroma of tendon sheath, desmoid‐type fibromatosis, lipoma, and other mineralized soft tissue neoplasms. The combination of a fat‐containing mass with internal mineralization is nonspecific on imaging and did not permit a definitive preoperative diagnosis.

**Figure 2:**
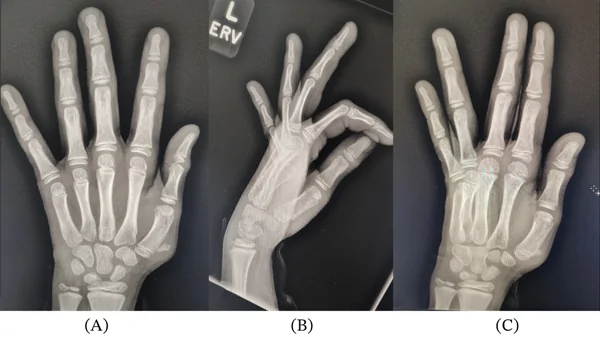
(A) Anteroposterior, (B) lateral, and (C) oblique radiographs of the left hand demonstrating a calcified soft tissue mass adjacent to the thumb metacarpal (white arrow).

**Figure 3:**
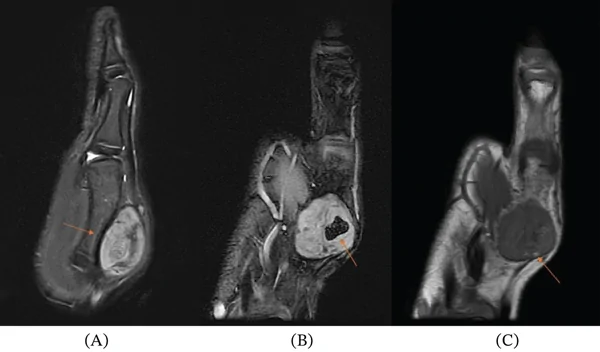
(A) MRI evaluation of the left hand. Sagittal STIR sequence showing a heterogeneous signal mass with hypointense capsule abutting the dorsal cortex of the first metacarpal. (B) There is concave remodeling of the metacarpal margin without local invasion or reactive marrow edema. Coronal gradient echo (GRE) sequence demonstrates lobular central hypointensity (arrow) matching the shape of the calcification seen on comparison radiograph. (C) Coronal T1 sequence demonstrates a heterogeneous signal mass mostly isointense to muscle, with small foci of T1 hyperintense macroscopic fat (arrow).

After discussion of the imaging findings and treatment options with the patient and family, the decision was made to proceed with excisional biopsy. The mass was approached through the interval between the extensor pollicis longus and extensor pollicis brevis tendons. Intraoperatively, the mass was adherent to the periosteum of the dorsal first metacarpal but was readily elevated from it. There was no continuity with the underlying cortex and no cortical breach; the lesion was therefore classified as juxtacortical. In situ, the mass measured approximately 1.8 cm in length and 1.4 cm in width. It was freed from the dorsal periosteum and surrounding soft tissues and excised en bloc. The patient was immobilized in a thumb spica splint for 2 weeks and was subsequently allowed to return to regular activities following confirmation of wound healing.

The specimen was submitted to the Mayo Clinic for pathologic evaluation. On gross examination, the intact specimen measured 2.8 × 2.4 × 1.5 cm. These dimensions correspond closely to the intraoperative measurement in one plane (1.5 vs. 1.4 cm) and are modestly larger in a second plane (2.4 vs. 1.8 cm); the intraoperative figures were in situ estimates obtained in two planes before the lesion was fully mobilized, whereas the gross measurement was obtained in three planes on the excised specimen, and the two are therefore not directly comparable.

Histologic sections stained with hematoxylin and eosin demonstrated a circumscribed lesion composed predominantly of lobules of mature adipose tissue, within which there were discrete foci of mature hyaline cartilage and lamellar bone (Figure 4). The adipocytes were uniform in size and shape, without nuclear atypia, hyperchromasia, or lipoblasts, and no mitotic activity, necrosis, or infiltrative growth was identified. Additional histologic parameters, including capsular status, the presence of a cartilage cap or endochondral ossification, intratrabecular marrow elements, and margin status, were not separately characterized in the referral pathology report; this is acknowledged as a limitation of the present report. The constellation of mature adipose, cartilaginous, and osseous tissue established the diagnosis of OCL.

**Figure 4:**
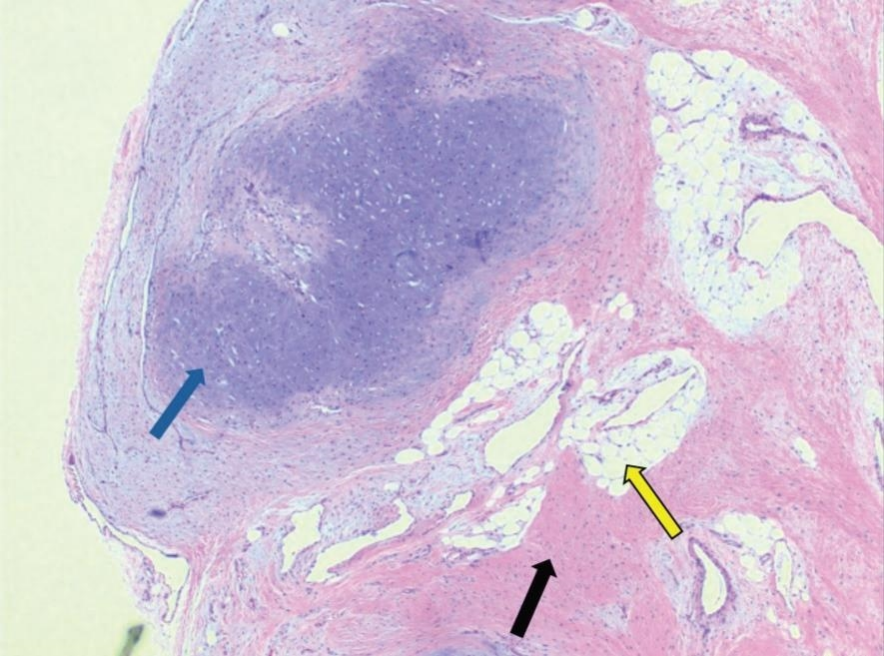
Photomicrograph of the excised specimen demonstrating mature adipocytes (yellow arrow) with osseous differentiation (black arrow) and cartilaginous differentiation (blue arrow); hematoxylin and eosin stain.

## 4. Discussion

Lipomas are benign tumors composed of mature adipocytes and represent one of the most common soft tissue neoplasms. They may arise in virtually any anatomic location and typically present as slow‐growing, painless masses. Lipomas can undergo differentiation into various histologic components, including fibrous tissue, blood vessels, muscle, bone, and cartilage [3]. OCL, also termed chondro‐osseous lipoma in some reports, is one of the rarest of these differentiated variants. A 2017 case report of a mandibular OCL included a literature review identifying only.

Fourteen previously reported cases across all anatomic sites, with approximately equal distribution among the maxillofacial region, the extremities, and the trunk, all occurring in adult patients [1]. A subsequent review accompanying a 2024 report of an OCL of the foot placed the cumulative total at 18 cases, with a reported mean patient age of 57.4 years and no clear sex predilection [2]. Involvement of the appendicular skeleton has been documented in adults, including lesions of the hand and of the foot and ankle [2, 4, 5]. Reports in the pediatric population are exceedingly rare; the pediatric case we identified was an intratentorial OCL in a 9‐year‐old male [3]. We make no claim of primacy, and the true incidence of this lesion in children cannot be estimated from the available literature. The relevant point for practice is that OCL can arise adjacent to bone in the hand of a skeletally immature patient and should be considered when a well‐circumscribed, mineralized, fat‐containing soft tissue mass is encountered.

Magnetic resonance imaging is the preferred modality for characterizing soft tissue lipomatous lesions, as lipid‐containing tissue produces characteristic homogeneous fatty signal intensities [6]. GRE imaging, sometimes referred to as black‐bone imaging, renders cortical bone hypointense while highlighting surrounding structures, allowing correlation of ossified foci within the mass with calcifications identified on plain radiography (Figures 2 and 3) [7]. These imaging features, in combination with histologic confirmation, are essential for establishing the diagnosis of OCL.

An important intraoperative finding in this case was the relationship of the lesion to the underlying bone. Although the mass was adherent to the periosteum of the first metacarpal, it was readily elevated from the periosteal surface and demonstrated no continuity with the underlying cortex; we therefore considered the lesion juxtacortical. This is notable because some reported OCLs demonstrate a parosteal origin with continuity to the underlying cortex, whereas others are juxtacortical and separable from the bone surface [5, 8]. In the present case, there was no cortical breach, no periosteal reaction, and no marrow involvement on imaging or at the time of surgery, and the lesion is therefore best characterized as juxtacortical. This distinction is relevant both for surgical planning and for classifying the lesion within the spectrum of bone‐associated soft tissue tumors.

The preoperative differential was necessarily broad because it rested on imaging alone. A well‐circumscribed, partially mineralized mass adjacent to the metacarpal shares overlapping radiographic features with tenosynovial giant cell tumor, fibroma of tendon sheath, and desmoid‐type fibromatosis, none of which can be excluded on imaging. The postoperative diagnosis was identified by the presence of mature adipose, cartilaginous, and osseous tissue on histology; this combination is not characteristic of the other entities in the preoperative differential and is diagnostic of OCL [9]. The differential therefore narrowed from a broad, imaging‐based list to a single histologically confirmed diagnosis only after excision.

The patient was seen in follow‐up at approximately 10 weeks postoperatively. At that visit, he reported no pain or discomfort. Range of motion and function of the extensor pollicis longus and extensor pollicis brevis tendons were intact and symmetric. There was no clinical evidence of local recurrence on physical examination. Recurrence was assessed clinically; surveillance imaging was not obtained. Longer‐term follow‐up data are not yet available, and given the limited number of reported OCL cases overall, the natural history and recurrence risk of this entity in pediatric patients remain uncertain.

In summary, this case demonstrates that OCL can arise adjacent to bone in the hand of a skeletally immature patient. Clinicians evaluating slow‐growing, painless soft tissue masses in children should consider OCL in the differential diagnosis regardless of patient age. Complete marginal excision provided both symptomatic relief and diagnostic confirmation in this patient; however, a single case with 10 weeks of follow‐up cannot establish the reliability of this approach, and definitive conclusions regarding recurrence risk await larger series and longer follow‐up.

## Funding

No funding was received for this manuscript.

## Conflicts of Interest

The authors declare no conflicts of interest.

## Data Availability

The data that support the findings of this study are available on request from the corresponding author. The data are not publicly available due to privacy or ethical restrictions.
